# Multi-level analyses of associative recognition memory: the whole is greater than the sum of its parts

**DOI:** 10.1016/j.cobeha.2020.02.004

**Published:** 2020-04

**Authors:** Gareth RI Barker, Elizabeth Clea Warburton

**Affiliations:** School of Physiology, Pharmacology andNeuroscience University of Bristol University Walk, Bristol BS8 1TD, United Kingdom

## Abstract

Associative recognition memory depends on the integration of information concerning an item and the spatio-temporal context in which it was encountered. Such an integration depends on dynamic interactions across a brain-wide memory network. Here we discuss evidence from multiple levels of analysis, behavioural, cellular and synaptic which demonstrating the existence of multiple overlapping, subnetworks embedded within these large-scale networks. Recent advances have revealed that of these subnetworks, a distinct hippocampal-prefrontal networks are engaged by different representations (object-spatial or object temporal). Other subnetworks are recruited by distinct processing demands, such as encoding and retrieval which are supported by distinct cellular and synaptic processes. One challenge to multi-level investigations of memory continues to be that conclusions are drawn from correlations of effects rather than from direct evidence of causation.

## Highlights

• Behavioural and systems, cellular and synaptic levels of analyses reveal multiple brain regions contribute to associative recognition memory.
• The key brain regions include the perirhinal and prefrontal cortices, thalamus and hippocampus
• Associative recognition memory depends on dynamic interactions between these regions.
• Distinct subnetworks are recruited for the processing of spatial and temporal information.
• The prefrontal cortex acts as a key hub for encoding and retrieval of associative recognition memory information.

**Current Opinion in Behavioral Sciences** 2020, **32**:80–87

This review comes from a themed issue on **Understanding memory: Which level of analysis?**

Edited by **Morgan Barense** and **Hugo J Spiers**

For a complete overview see the Issue and the Editorial

Available online 17th March 2020

**https://doi.org/10.1016/j.cobeha.2020.02.004**

2352-1546/© 2020 The Authors. Published by Elsevier Ltd. This is an open access article under the CC BY license (http://creativecommons.org/licenses/by/4.0/).

## Introduction

Recognition of an object, such as your car in a carpark can be achieved by a judging the familiarity of the car’s features (shape, model colour). However importantly recognition can be greatly facilitated by also remembering where it was parked in relation to other surrounding stimuli, such as a tree, building or sign, that is, associated spatial information or by remembering when it was parked, that is, associated temporal information. Studies of recognition memory, at systems, cellular, and synaptic levels of experimental analysis have revealed that single item and associative recognition are mediated by distinct neural substrates. Thus, in the case of single item recognition behavioural studies have shown that such recognition is associated with differential neuronal activation in the perirhinal cortex PRH [1] and is impaired by PRH damage [2]. Investigations at a cellular level have shown that PRH neurons signal the familiarity of individual stimuli by reductions in neuronal activity and evidence at a synaptic and computational level show that these response decrements can be mediated by synaptic weakening [3,4]. Thus, the PRH is the site of storage for information necessary for recognising single items. Associative recognition, that is, for remembrance of the car and surrounding stimuli, on the other hand requires the integration of different types of information (item, spatial, contextual, temporal). Consequently, evidence has shown that this form of recognition memory engages multiple brain regions including the prefrontal cortex (PFC), areas within the medial temporal lobe (PRH, hippocampus (HPC), entorhinal cortex (EC)) and distinct thalamic nuclei, and that these regions operate within neural circuits. Here, evidence from human and rodent studies across behavioural and systems, cellular and synaptic levels of analysis will be presented (note: there is also consistent evidence from non-human primate studies not presented here due to space constraints). The review will explore how these networks operate, and how information may be relayed within a brain-wide circuit to support the processing of associative recognition memory information.

## Investigations at a behavioural and systems level

Functional magnetic resonance imaging (fMRI) studies using different stimuli, protocols and analyses have demonstrated that successful associative memory performance is correlated with increased activation within the medial temporal lobe (HPC, parahippocampal cortex), PFC and thalamus [5, 6, 7, 8] and coordinated activity between brain regions reveal task-related functional connectivity [9,10] across highly distributed brain-wide associative memory networks. The spatio-temporal dynamics of fMRI studies have enabled an examination of the neural regions and networks involved in memory encoding and retrieval. Such studies have shown that regional patterns of activity associated with encoding are reinstated during successful retrieval [9], but recently an analysis of network connectivity during encoding and retrieval also demonstrated that depending on the type of associative information (i.e. spatial or temporal) successful retrieval required flexibility in the operation of neural networks involved [11].

Consistent with the imaging data lesion studies in humans have shown that damage in the PRH, EC parahippocampal cortices, HPC, PFC and thalamus impairs associative recognition memory [12, 13, 14]. However, depending on the location of the lesion, the precise nature of the cognitive deficit varies. For example, damage in the PRH has been reported to impair associative memory by disrupting item memory, stimulus unitization [15,16] or complex object discriminations [17]; HPC damage to impair the integration of different types of information [18^•^] or relational memory [19] while damage in the PFC appears to impair decision making and/or their use of strategies for associative memory retrieval [20]. However, in patients, damage is rarely restricted to a single brain area, thus understanding memory processes solely from these studies is limited and generalising observations from small numbers of patients where damage is anatomically restricted is also problematic. Hence a clear benefit may be derived from testing memory in animals with focal lesions using tasks that appropriately model human recognition memory processes.

**Figure 1:**
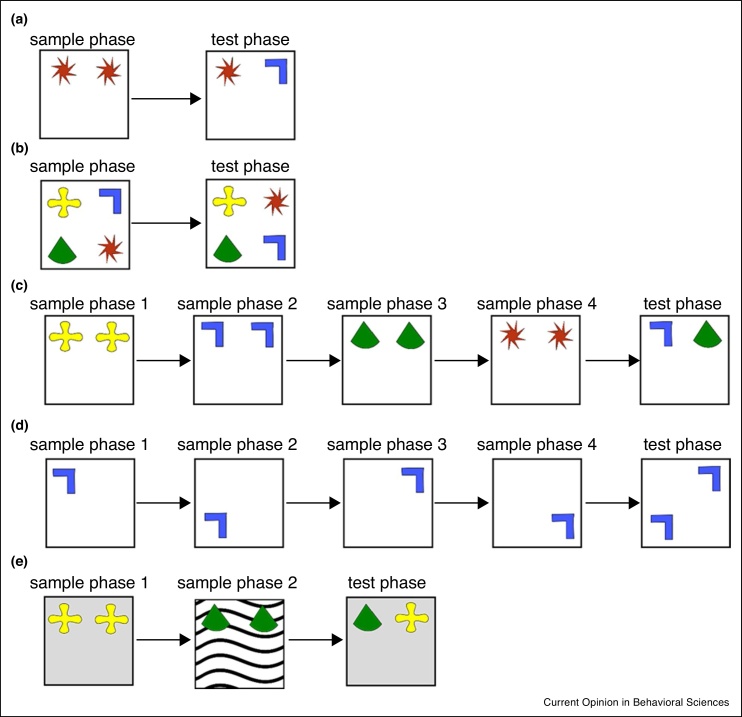
Spontaneous preferential exploration tasks in rodents. **(a) Novel object recognition task**, in the sample phase two identical objects are presented. In the test phase a novel object replaces one of the sample objects. **(b) Object-in-place task**, four different objects are presented in the sample phase. In the test phase, two objects switch location and object-in-place memory is demonstrated by preferential exploration of the rearranged objects. **(c) Temporal order memory task**, a sequence of objects is presented in four sample phases. In the test phase animals two objects are presented and intact memory is demonstrated by a preference for the object presented earlier in the sequence. **(d) Temporal location task**, the same object is presented in a sequence of different locations. In the test phase memory is demonstrated by preferential exploration of the object in the location occupied earlier in the sequence. **(e) Object-in-context task**. In sample phase 1 objects are presented in one context, in sample phase 2 different objects are presented in a different context. In the test phase one object is ‘in context’, while the other object is ‘out of context’ and intact object-in-context memory is demonstrated by preferential exploration of the ‘out of context’ object.

Spontaneous preference tests, also called object recognition tests, have been widely used, in our lab and others, to assess recognition memory in rats and mice. These tasks, which rely on an animals’ innate preference for novelty in the environment, typically consist of two phases, a ‘sample phase’ in which the animal explores objects, and a ‘test phase’. To measure associative recognition memory two objects from the sample phase exchange locations in the test phase, and the time spent exploring the novel compared to the familiar object-place configuration is used to measure memory performance (for some variants of this task see Figure 1). These tasks have the advantage of mapping closely onto preferred-viewing tests of human recognition memory [21,22]. Further in contrast to other memory tasks, such as the delayed non-matching to sample task, these tests of object recognition are not dependent on reinforcement, thus do not require extensive training and are not impacted by manipulations that affect motivation or rule learning [21,23,24].

Using these object recognition tasks lesions in the PRH [1,25,26], HPC [26,27] medial PFC (mPFC; specifically the infralimbic and prelimbic cortices) [25,26,28, 29, 30], lateral EC [31], postrhinal cortex [32], mediodorsal nuclei of thalamus (MD) [29], nucleus reuniens of thalamus (NRe) [33] produce deficits across multiple tasks (object-in-place, temporal order or object-in-context). Thus common cellular mechanisms maybe engaged for both object-spatial/contextual and object-temporal associations. These studies, the results of which are consistent with human studies, do not indicate potential cellular mechanisms or the importance of regional interactions in memory formation.

**Figure 2:**
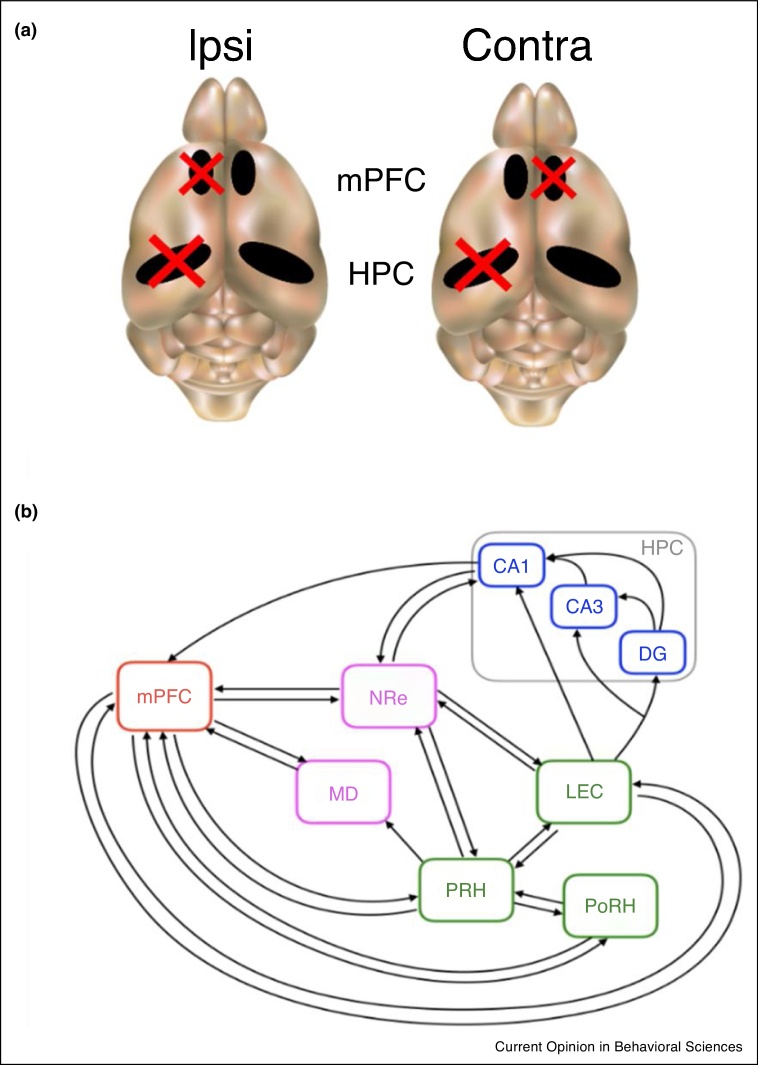
**(a)** Schematic showing the principle of disconnection technique, where the red cross indicates the placement of a lesion in the HPC or mPFC in the same hemisphere (Ipsi) or opposite hemispheres (Contra). **(b)** Schematic of the associative recognition memory network identified by lesion and disconnection studies. Major anatomical connections between regions are shown only. (HPC hippocampus; DG dentate gyrus mPFC medial prefrontal cortex; LEC lateral entorhinal cortex; PRH perirhinal cortex; PoRH postrhinal cortex; NRe nucleus reuniens of the thalamus; MD medial dorsal nucleus of the thalamus).

One method which can be used to examine whether specific brain areas operate within a memory circuit is a disconnection analysis. This approach involves placing unilateral lesions in two different brain regions, for example the PFC and HPC in opposite hemispheres. If this disconnection has a significant effect on behaviour, compared to unilateral lesions placed in the same hemisphere, it is concluded that the two brain regions form part of a functional system [24,25, Figure 2a]. Using this technique, disconnection of pairs of structures including the HPC, PFC, other medial temporal lobe regions (PRH, lateral EC, postrhinal cortex) and MD [24,25,29,34, 35, 36] has been shown to significantly impair object-in-place, temporal order, and object-in-context recognition memory, consistent with the existence of a brain-wide associative recognition memory network (Figure 2b). However, these studies only rely on the temporary or permanent structural inactivation and do not provide insights into network dynamics, routes or directionality of the interaction during memory formation. As brain regions are directly, indirectly and reciprocally interconnected (Figure 2b) more specific projection targeting manipulations, such as optogenetics or pharmacogenetics combined with anterograde or retrograde viruses have been used.

**Figure 3:**
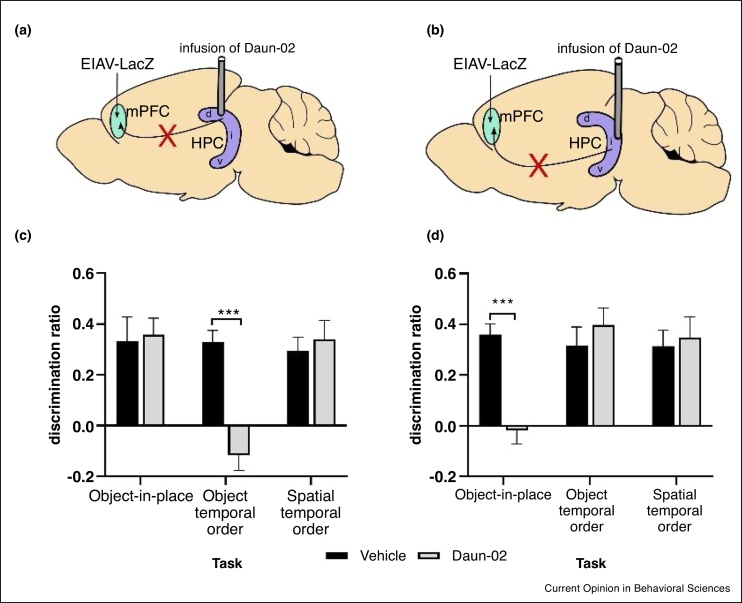
Deactivation of anatomically distinct projections from CA1 to mPFC reveals contrasting roles in associative recognition memory. **(a)** and **(b)** Strategy used to deactivate direct CA1 → mPFC projections. A pseudo-rabies coated lentiviral vector expressing Lac-Z (EIAV-LacZ) injected into medial prefrontal cortex (mPFC), and transported retrogradely to the soma of neurons projecting to mPFC. Cannulae were implanted bilaterally either over the dorsal CA1 (dCA1,) or intermediate CA1 (iCA1,). The prodrug Daun-02 is infused through cannula into the HPC where it is converted into daunorubicin resulting in selective deactivation of the CA1 → mPFC. **(c)** Deactivation of dCA1 → mPFC significantly impairs temporal order memory not object-in-place or temporal location. **(d)** Deactivation of iCA1 → mPFC significantly impaired object-in-place task but not temporal order or temporal location. Memory performance expressed as mean discrimination ratio (time exploring novel object-time exploring familiar object/total exploration) ±sem. *** *p* < 0.001. Adapted from Ref. [32].

One study [37^••^] investigated the importance of the direct projection between separate regions of the CA1 hippocampal subfield and mPFC (CA1 → mPFC) in associative memory for object-spatial or object temporal information. Using a retrograde-pharmacogenetic approach (Figure 3a;b) they were able to target populations of CA1 → mPFC neurons arising in the dorsal or the intermediate region of the CA1 and found that deactivation of the dorsal CA1 → mPFC projection selectively impaired object temporal order memory (Figure 3c). In contrast, deactivation of the intermediate CA1 → mPFC impaired object-in-place memory, but was without effect on temporal order memory (Figure 3d). Neither projection was required for spatial temporal order memory (Figure 3c;d). Thus, functionally distinct HPC-mPFC subnetworks appear to mediate different recognition memory processes [37^••^]. This complex segregation of function within a memory network has not only been observed in projections to the mPFC. A recent study [38^••^] tested the role of output pathways from mPFC to the NRe (mPFC → NRe) and perirhinal cortex (mPFC → PRH). Both pathways were necessary for memory performance, but the mPFC → NRe pathway was crucial for working memory retrieval strategies whereas the mPFC → PRH pathway was crucial for a temporal context retrieval strategy [38^••^]. Integration of these findings and the results from lesion studies in rats [24,25,33] and patients [19], together with recent studies describing the complex organisation of the networks between the mPFC and thalamic nuclei [39] indicate that the medial prefrontal cortex is a key hub for both the formation, integration and retrieval of associative recognition memory information.

## Investigations at a cellular level

*In vivo* electrophysiological studies enable an examination of neuronal signatures of associative memory. In humans, intracranial EEG (iEEG) recordings from electrodes positioned subdurally or implanted into the medial temporal or frontal lobe of epilepsy patients have examined activity of individual neurons and neural oscillation across multiple frequency bands during associative memory encoding and retrieval using word-colour association tasks, item-place task (i.e. navigating a virtual environment to deliver ‘items’ to a precise location) or a verbal paired associate tasks [40, 41, 42]. These studies showed that patterns of HPC and cortical activity observed during memory encoding were reinstated during memory retrieval but within a compressed timescale, and that retrieval-related activity occurs first in the HPC in a processes of pattern completion, before the reinstatement of activity in the cortex (for a recent review see Ref. [40]). While the high temporal and spatial resolution offered by the iEEG technique are of great value to elucidating associative memory mechanisms, the range of brain regions that have been examined are limited by the fact that the placement of electrodes is determined by clinical need. In animals, neuronal firing in the PRH, mPFC, lateral EC and HPC are modulated by objects and object place associations [43,44]. While neural spiking in different regions appear to correspond to distinct features of the task, the firing patterns across regions are temporally coordinated suggesting that associative memory is dependent on functional interactions between brain regions at specific time points [45,46].

To explore, in detail, the brain-wide memory networks of recognition memory, high resolution imaging of immediate early gene (IEG) expression in rodents has been used. IEGS, such as *c-fos* and *Arc*, are readily expressed in brain regions, following learning and importantly can reveal activation within anatomical subregions, different cortical layers and even different cell types. While IEGs are indirect markers for neuronal activity, both *c-fos* and *Arc* have been linked to synaptic plasticity processes, such as long-term depression (LTD) and long-term potentiation (LTP) associated with recognition memory [47,48]. Hence expression patterns of these IEGs may provide direct evidence of underlying cellular mechanisms of memory formation.

IEG imaging has been used to map neural activation across multiple dimensions of memory processing. Previous studies have compared activation to novel versus familiar stimuli or novel versus familiar configurations of stimuli, or object-spatial versus non-spatial and object temporal order information. In one study the presentation of novel object-place configurations produced greater *c-fos* activation in area CA1 and postrhinal cortex, while familiar object-place configurations produced greater *c-fos* activation in the area CA3 [49]. Imaging of *c-fos* has also been combined with behavioural and computational analyses, such as structural equation modelling, to correlate changes in activity between brain regions after learning and produce models of ‘best fit’. Application of structural equation modelling to *c-fos* expression data acquired after animals actively explored sequences of novel or familiar objects revealed neural network models containing separate processing pathways for novel and familiar information; a ‘novel’ processing pathway from the lateral EC to the dentate gyrus and CA3 via the perforant pathway, and a ‘familiar’ processing pathway from lateral EC to CA1 via the temporammonic pathway [50^•^,^51^]. In another study, the high spatial resolution of this cellular imaging technique enabled an examination of the involvement of the CA1 and CA3 subfields across the proximodistal axis of the HPC in relation to object-place and temporal order memory [52,53^•^]. By imaging *Arc* mRNA, Beer and colleagues reported that neurons in distal CA1 were tuned to temporal information, whereas proximal CA1 and CA3 neurons were tuned to spatial information. This pattern of neural activation parallels the topographical representation of object temporal and object-spatial information revealed in behavioural studies [37^••^] and by combining across different levels of analyses one can begin to understand the complexities and intricacies of memory subnetworks within the medial temporal lobe, and between the medial temporal lobe and PFC.

## Investigations at a synaptic and molecular level

The imaging, lesion and electrophysiological techniques described so far offer evidence of the structure of and neural correlates of memory networks, but further insight into the mechanisms of information processing and storage may be achieved by identifying the cellular mechanisms that within specific brain regions. One way is to manipulate synaptic plasticity and examine the effects of such manipulations on memory performance. For example, it has been shown that induction of LTP and LTD in the HPC and PRH is inhibited by application of the competitive NMDA receptor antagonist AP5, [48,54]. In behavioural studies infusion of AP5 into the PRH impairs object recognition, and infusion of AP5 into the PRH, HPC or mPFC impairs object-in-place memory [55]. Interestingly there also appears to be a strong correlation between the mechanisms of object recognition memory and associative recognition memory within PRH [56,57] but while the pharmacological studies provide evidence linking synaptic plasticity mechanisms with behaviour, the effects of drugs like AP5 on memory could be mediated by an LTP-like or an LTD-like mechanism. Hence in a recent study, we examined whether it was possible to dissociate the contribution of LTP and LTD in the mPFC to recognition memory. This study looked specifically at the role of cholinergic neurotransmission via distinct nicotinic receptor subtypes in the mPFC. We found that nicotinic α7 receptors were crucial for the induction of LTP but not LTD, and blockade of nicotinic α7 receptors selectively impaired object-in-place memory encoding. In contrast nicotinic α4β2 receptors were crucial for the induction of LTD but not LTP and blockade of these receptors impaired object-in-place memory retrieval but was without effect on encoding [58^•^]. Thus, combining studies at synaptic and behavioural levels of analysis can reveal the cellular mechanisms of memory formation, and how these relate to the different stages of memory processing.

## Conclusions

Our conceptualization of associative recognition memory formation derives from evidence provided by different levels of experimental analysis (behavioural, cellular computational, synaptic, molecular) and by synthesising information derived from both humans and animals. Together studies have revealed the existence of multiple medial temporal lobe-prefrontal cortex memory networks the operation of which is determined by the ongoing cognitive processing demands. Behavioural studies non-invasive imaging and lesion studies show the necessity of brain regions operating within brain-wide memory networks, and *in vivo* recording, synaptic and molecular techniques enable measurement of the neural subprocesses that underpin memory formation. Combining evidence across levels of analysis provides the spatio-temporal resolution required for a detailed dissection of associative memory networks. Successful encoding depends on neurons in the PRH coding object identity, item information and relative familiarity, while in the HPC information related to the spatial and/or temporal context is acquired within segregated subnetworks. This item-context information is integrated within the network via direct CA1-mPFC projections. Retrieval is mediated by projections from the mPFC to the thalamus and medial temporal lobe. Thus the mPFC emerges as a key associative memory node and indeed encoding and retrieval of associative recognition memory representations depend on different synaptic plasticity mechanisms in the mPFC. One challenge to multi-level investigations of memory continues to be that conclusions are drawn from correlations of effects rather than from direct evidence of causation. Technological advances such as the development of mouse lines with cell type specific expression of CRE-recombinase, has allowed a more direct examination of how memories may be organised. In addition, the development of mouse lines where neurons active within a specific time window can be labelled, referred to as engram cells, allows examination of the properties and functions of cells activated during a specific learning event [59]. Application of these new techniques, combined with precise behavioural protocols that map human memory, as well as the increased anatomical and temporal specificity afforded by high resolution fMRI and iEEG, will allow a deeper understanding of both the micro-circuit and macro-circuit function of the associative recognition memory network.

## Conflict of interest statement

Nothing declared.

## References and recommended reading

Papers of particular interest, published within the period of review, have been highlighted as:

• of special interest
•• of outstanding interest

## CRediT authorship contribution statement

**Gareth RI Barker:** Data curation, Methodology, Validation, Visualization, Investigation, Project administration. **Elizabeth Clea Warburton:** Conceptualization, Funding acquisition, Investigation, Resources, Project administration, Supervision, Visualization, Writing - original draft, Writing - review & editing.
